# *Porphyromonas gingivalis* lipopolysaccharide induced RIPK3/MLKL-mediated necroptosis of oral epithelial cells and the further regulation in macrophage activation

**DOI:** 10.1080/20002297.2022.2041790

**Published:** 2022-02-27

**Authors:** Fengxue Geng, Junchao Liu, Chengcheng Yin, Shuwei Zhang, Yaping Pan, Hongchen Sun

**Affiliations:** a Department of Periodontics, School and Hospital of Stomatology, China Medical University, Shenyang, China; b Center of Implant Dentistry School and Hospital of Stomatology, China Medical University, Shenyang, China; c Department of Periodontics and Oral Biology, Liaoning Provincial Key Laboratory of Oral Diseases, School and Hospital of Stomatology, China Medical University, Shenyang, China; d Department of Oral Pathology, China Medical University School of Stomatology, Shenyang, China

**Keywords:** *Porphyromonas gingivalis*, oral epithelial cells, necroptosis, DAMPs, macrophage polarization

## Abstract

Necroptosis, a new type of regulated cell death with massive release of damage-associated molecular patterns (DAMPs), is involved in the pathogenesis of periodontitis. However, the role of necroptosis in oral epithelial cells and the following effect on macrophages activation remain unknown.

Human immortalized oral epithelial cells were stimulated with *Porphyromonas gingivalis* lipopolysaccharide (LPS). Cell death was assessed while expressions of RIPK3/MLKL and toll-like receptors (TLRs) were evaluated. Necrosulfonamide (NSA), an inhibitor of MLKL was applied to block necroptosis. The expression of DAMPs and the epithelial connection protein were evaluated by qPCR and immunofluorescence, respectively. Immortalized human monocytes U937 were induced into the M0 or M2 subset, and influences of HIOECs-derived DAMPs on macrophage polarization as well as activation of the Mincle/SYK axis were assessed.

*P. gingivalis* LPS could be recognized by TLR2 and regulates necroptosis of HIOECs by activating RIPK3/MLKL. NSA inhibited cell death of HIOECs, alleviated impaired epithelial connection, and inhibited expressions of DAMPs. Low dose of DAMPs derived from HIOECs promoted M2-like polarization by activating the Mincle/SYK axis, which was significantly suppressed with increased doses of DAMPs.

*P. gingivalis* LPS destructed oral epithelial cells via RIPK3/MLKL-mediated necroptosis, which further regulated macrophage activation via DAMPs from oral epithelial cells.

## Introduction

Periodontitis, a chronic inflammatory condition with complicated pathogenic factors, is one of the most common oral diseases that results in the destruction of tooth supporting tissues. According to a recent comparative study from 1990 to 2017, severe periodontitis is regarded as a serious burden in Asian countries, especially in China and India [1]. Therefore, it is urgently needed to raise the awareness of periodontal health and to adopt therapeutic measurement at the initial stage of periodontitis.

Oral epithelial tissue is the first physical and immunological barrier against periodontal infections. The destruction of epithelial connection and the loss of oral epithelial cells will increase the possibility of periodontal pathogen invasion, which is considered as an important step in the initiation and development of periodontitis [1–3]. Recently, necroptosis, a newly regulated necrosis mediated by the receptor-interacting protein serine-threonine kinases-3 (RIPK3)/mixed lineage kinase domain-like protein (MLKL), is a novel pathogenic mechanism of periodontitis [4–6]. *Porphyromonas gingivalis* is a keystone periodontal pathogen with various virulence factors including lipopolysaccharide (LPS) and gingipains [7]. It was reported that *P. gingivalis* induced cellular necroptosis in monocytes, periodontal ligament fibroblasts (PDLFs) as well as periodontal ligament stem cells (PDLSCs) [8–10]. NSA, a small molecule inhibitor specific to MLKL, was reported to block necroptosis and promisingly applied in various diseases such as amyotrophic lateral sclerosis and Alzheimer’s disease [11]. However, to the best of our knowledge, the role of *P. gingivalis* in necroptosis of the oral epithelial barrier and the possible effect of NSA remains unknown.

Although cell death was initially regarded as the result of inflammation, it is recently considered that certain types of cell death may amplify the inflammatory response [12]. Unlike other types of cell death such as apoptosis with limited damage-associated molecular patterns (DAMPs) release [13], necroptosis can induce massive release of immunomodulatory factors such as DAMPs, which may be recognized by pattern recognition receptors (PRRs) and thereafter aggravates immune responses by continuously inducing expression of chemokines and cytokines [13,14]. It is known that the inflammatory reaction is modulated via macrophages (Mφs) in various diseases by specific DAMPs, such as basic calcium phosphate (BCP) crystals, calcium-binding protein S100A8/A9 and Human β-defensin 3 [15–17]. Mφs belong to the frontline defense cells of the human immune system, which have dual roles in the inflammation based on the concept of Mφ polarization. Mφs differentiate into the M1 pro-inflammatory phenotype, which produces inflammatory factors such as IL-6, and the M2 anti-inflammatory phenotype, which secretes cytokines such as IL-10 for wound healing and tissue repair [18]. In recent years, it has been widely accepted that Mφ polarization in gingival tissue is responsible for the development and progression of periodontal tissue destruction [19]. Furthermore, reduction of the M1/M2 ratio could inhibit alveolar bone loss in mouse periodontitis models [20]. Thus, understanding the roles of DAMPs released from epithelial cells during Mφ polarization will not only help to understand the cross-talk between oral epithelial cells and Mφs but also could provide a new approach to prevent and treat periodontitis.

The Mφ-inducible Ca^2+^-dependent lectin receptor (Mincle), which is mainly expressed by myeloid cells such as Mφs, is a primary PRR of the innate immune response that senses DAMPs [21]. Previous studies have demonstrated that Mincle and its downstream spleen tyrosine kinase (SYK) signaling are activated in several inflammation-related diseases [22]. For instance, a recent study demonstrated that Mincle/SYK promote the inflammatory response by upregulating the M1 Mφ activity, which reveals a novel target to alleviate DAMPs-related acute kidney injury [23]. On the contrary, the anti-inflammatory role of Mincle was also revealed not only in cytokine secretion but also in the inhibition of pro-inflammatory signals [24,25]. We hypothesized that oral epithelial-derived DAMPs within the periodontal microenvironment may be also sensed by Mincle and participate in the regulation of Mφ polarization.

For the first time, the study herein explored the possible effects of necroptosis in oral epithelial cells by *P. gingivalis* LPS stimulation. NSA was used to block necroptosis of oral epithelial cells, which might be applied as a promising target for the front line of defense against periodontal infection. We also revealed a novel mechanism of oral epithelial cells-derived DAMPs in the regulation of Mφ activation.

## Materials and methods

### Cell cultures

HIOECs were kindly provided by Professor Wantao Chen from the Key Laboratory of Shanghai Oral Medicine, Ninth People’s Hospital, Shanghai Jiao Tong University. The human monocyte U937 cell line was obtained from ATCC (Manassas, VA). HIOECs were cultured with Defined Keratinocyte-SFM (GibcoTM, Thermo Fisher Scientific Inc., MA) plus growth supplement. The Monocyte U937 cell line was cultured with RPMI 1640 medium (Thermo Fisher Scientific) with 10% fetal bovine serum (Thermo Fisher Scientific) plus penicillin-streptomycin (Thermo Fisher Scientific) at 37°C in an incubator with humidified atmosphere containing 5% CO_2_.

To evaluate the effects of *P. gingivalis* on necroptosis of oral epithelial cells, HIOECs were cultured with *P. gingivalis* (LPS, InvivoGen, CA) at 0.1 to 1 µg/mL. For the blocking assay, HIOECs cells were pretreated with a specific inhibitor of MLKL, NSA (Cayman Chemical, M), at certain concentrations for 2 h before LPS stimulation [10]. To induce differentiation of U937 into Mφ‐like cells, phorbol myristic acid (PMA) (Cayman Chemical) at 100 ng/ml was added into the culture for 48 h. To induce M2-like phenotypes, the medium was replaced with fresh medium with 5% fetal bovine serum plus 30 ng/mL of recombinant human IL-4 (PeproTech, NJ, USA) without PMA for another 24 h culture [26,27].

### Cell viability assay

The cell counting kit 8 (CCK-8, Dojindo, Kumamoto, Japan) was used to evaluate cell proliferation. HIOECs were plated at 3,000 cells/well in 96-well plates. *P. gingivalis* LPS at 0.1 to 1 µg/mL was added when the cells had reached 80% of confluence and cultured for 48 h, then CCK-8 solution (10%) was added and the cells were incubated at 37°C for 2 h. Optical density was measured at 450 nm using a microplate reader (Tecan, Untersbergstrasse, Grödig, Austria). The group without LPS was considered as negative control.

### Lactate dehydrogenase cytotoxicity assay

Intracellular lactate dehydrogenase (LDH) released into the culture medium through the rupture of the plasma membrane is regarded as a hallmark of cellular necrosis [4]. To test the role of *P. gingivalis* LPS in cell necroptosis, the cell culture supernatant was collected when HIOECs were stimulated by *P. gingivalis* LPS for 48 h. The LDH assay was performed according to the manufacturer’s instructions (Wanleibio, Shenyang, China). Absorbance was measured at 450 nm using a microplate reader. The activity of LDH was calculated based on the instruction. The group without LPS stimulation were considered as a negative control.

### Preparation of oral epithelial cells derived-DAMPs

Briefly, HIOECs were harvested by trypsinization, and 10^6^ cells were resuspended in 500 µL of RPMI 1640 medium, then did freezing-thawing were performed 5 times (which avoided interference of *P. gingivalis* LPS on Mφ polarization). The cells were spun at 400 × g at 4°C to collect the supernatant and were stored at −80°C for further use [14]. Before using DAMPs to stimulate, the culture medium of M0 or M2-like Mφs was replaced with fresh medium with 5% fetal bovine serum. Doses of DAMPs were calculated according to the ratio of cell numbers between HIOECs that released DAMPs and Mφ‐like cells [10,14].

### Quantitative real-time polymerase chain reaction (qPCR)

Total RNAs were extracted using RNAiso Plus, and cDNA was synthesized using the RR047A kit. The qPCR was performed using the RR820A kit with an Applied Biosystems 7500 Real-Time PCR System (Waltham, MA). All the reagents were obtained from Takara (Kyoto, Japan). The 2^−ΔΔ^CT method was applied to calculate gene expression fold changes [28]. Primer sequences are listed in Table S1.

### Western blot

Protein was extracted with the RIPA Lysis Buffer containing phenylmethane-sulfonyl fluoride (PMSF) and phosphatase inhibitor (PPI) with appropriate vibration on ice for 30 min. The protein concentration was determined using the BCA protein assay kit (Beyotime Biotech, Shanghai, China). From each group 50 µg was used to run SDS-polyacrylamide gel electrophoresis and transferred to the nitrocellulose membrane. After blocking in 5% skim milk in tris-buffered saline with 0.1% Tween (TBST) for 60 min at room temperature, blots were incubated with the following anti-rabbit primary antibodies, anti-MLKL (1:1,000, Abcam, Cambridge, MA), anti-p-MLKL (1:500, Abcam), anti-RIPK3 (1:500, Proteintech Group, Chicago, IL); anti-p-RIPK3 (1:1,500, Abcam), anti-TLR2 (1:500, Wanleibio, Shenyang, China), anti-TLR3 (1:500, Wanleibio), anti-TLR4 (1:500, Wanleibio), anti-iNOS (1:500, Abcam), anti-Arg-1 (1:1,000, Abcam), anti-Mincle (1:1,000, GeneTex, Irvine, CA), anti-SYK (1:500, Abcam, Cambridge, MA), anti-p-SYK (1:500, Cell Signaling Technology, MA) GAPDH (1:1,500, Bioworld, MN) or anti-α-Tubulin (1:1,500, Proteintech Group) at 4°C overnight. Membranes were washed with TBST three times, and incubated with goat anti-rabbit IgG IRDye1 800CW secondary antibody (LICOR, Lincoln, NE, USA) at room temperature for 1 h, washed with TBST three times, then incubated with substrate. Blots were detected with Odyssey CLX (LI-COR). Densitometric analysis was made with the Image J software (1.42q).

### Immunofluorescence staining

Cells were cultured on sterilized glass slides in 24-well plates, treated with pre-cooled paraformaldehyde (4%) for 30 min and Triton X-100 (0.1%) at room temperature for 10 min, blocked with 1% BSA for 30 min, then incubated with primary antibody at 4°C overnight, anti-MLKL (1:100, Abcam), anti-E-cadherin (E-cad) (1:500, Abcam), anti-CD86 (1:100, Abcam) and anti-CD206 (1:100, Abcam). Finally, slides were incubated with fluorescence labeled secondary antibody (1:1,000) for 1 h at room temperature, then stained with DAPI for 5 min, and mounted with antifade mounting medium. Slides were observed with a fluorescence microscope.

### Role of Mincle/SYK in periodontal regeneration via analyzing data from GEO

To further explore the role of Mincle/SYK in periodontal regeneration, bioinformatical data were acquired from the Gene Expression Omnibus (GEO) platform of NCBI. A study (GSE2525) was selected and analyzed with GEO2R, which contains microarray data analyzed using paired sets of human gingival and regenerating cells isolated from patients who underwent a regenerative surgical procedure.

### Statistical analyses

The results were shown as mean ± standard deviation (SD). Each experiment was repeated at least three times. Non-parametric tests were applied for the statistical analysis with the GraphPad Prism 8.0.1 software. Differences between two groups were analyzed with the Mann-Whitney U-test. *P* < 0.05 was considered statistically significant.

## Results

### P. gingivalis *LPS modulated necroptosis of oral epithelial cells*

**Figure 1.**
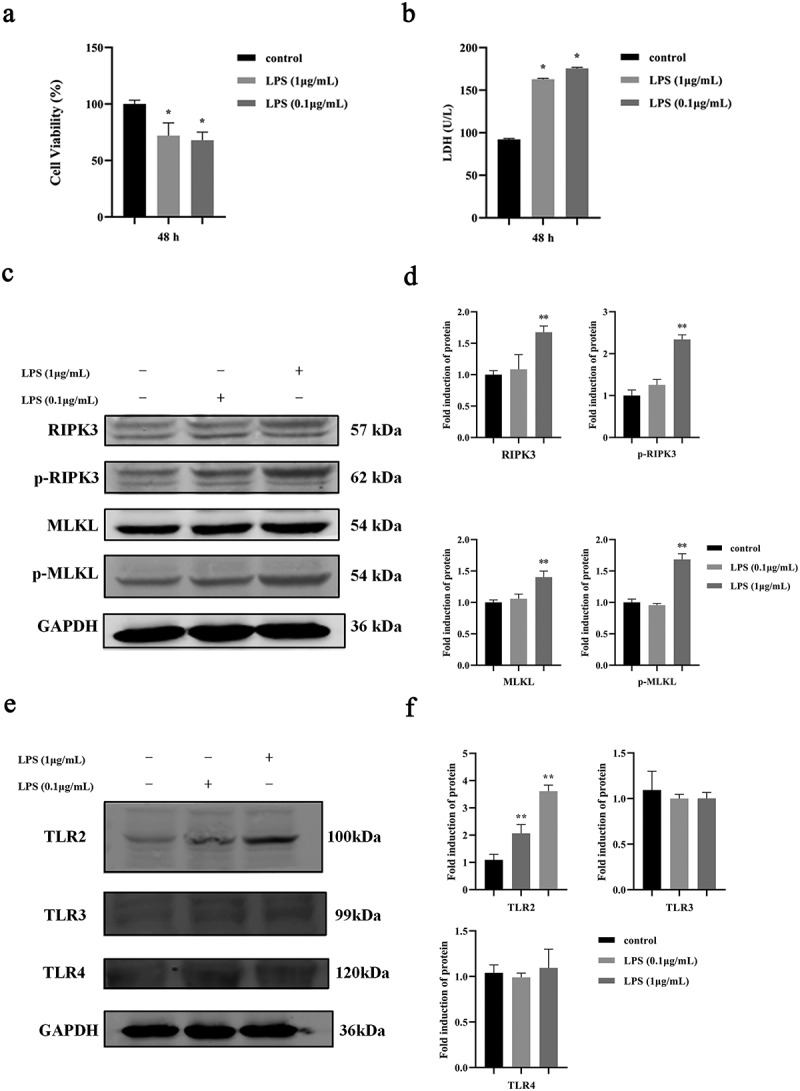
Effect of *P. gingivalis* LPS on necroptosis of oral epithelial cells. **a**. Cell proliferation of HIOECs cocultured with *P. gingivalis* LPS at 0.1–1 µg/mL for 48 h. **b**. Release of LDH from HIOECs cocultured with *P. gingivalis* LPS at 0.1–1 µg/mL for 48 h. **c, d**. Necroptosis-associated protein expression by Western blots and bar graphs of relative fold changes were shown when HIOECs were stimulated with *P. gingivalis* LPS at 0.1–1 µg/mL for 24 h. **e, f**. Western blots of TLR2, TLR3, TLR4 and GAPDH and bar graphs of relative fold changes were shown when HIOECs were stimulated with *P. gingivalis* LPS at 0.1–1 µg/mL for 24 h. *, *P* < 0.05, **, *P* < 0.01.

To explore if *P. gingivalis* infection can induce necroptosis of oral epithelial cells, HIOECs were co-cultured with *P. gingivalis* LPS. Data showed that HIOECs proliferation was inhibited with *P. gingivalis* LPS from 0.1 to 1 µg/mL (Figure 1a) while the LDH release was increased (Figure 1b). Important proteins involved in necroptosis, RIPK3, p-RIPK3, MLKL and p-MLKL were significantly upregulated at 1 µg/mL of *P. gingivalis* LPS (Figure 1c, d). This indicates that *P. gingivalis* LPS can induce necroptosis of oral epithelial cells at 1 µg/mL, a concentration applied in all the following experiments. It is known that TLRs are membrane-bound PRRs and are activated during *P. gingivalis* infection [6,14]. We found that the expression of TLR2 was upregulated while there was no significant difference in the TLR3 and TLR4 expressions (Figure 1e, f).

### *NSA blocked necroptosis of oral epithelial cells induced by* P. gingivalis *LPS and DAMPs release*

**Figure 2.**
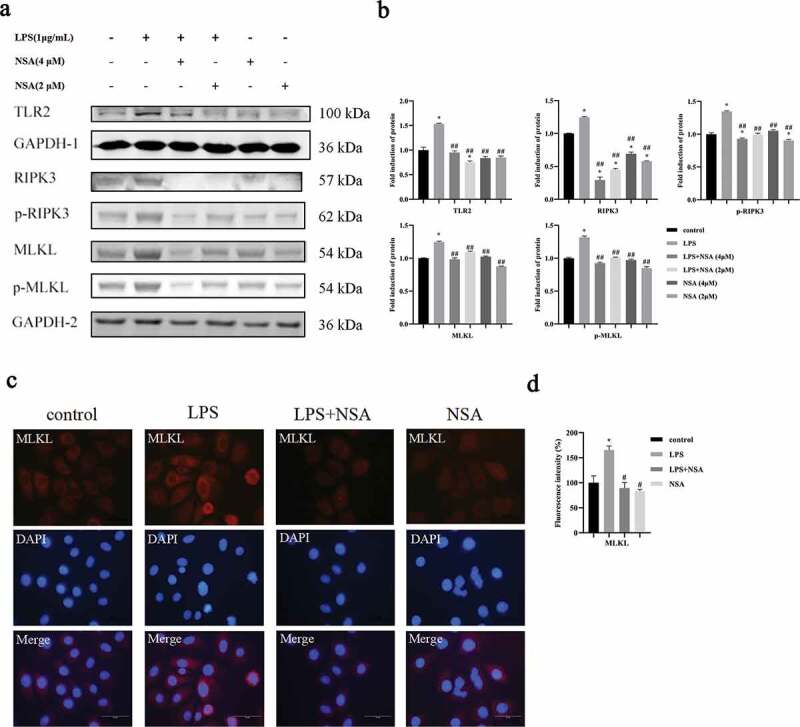
NSA inhibited the expression of necroptosis associated genes as well as TLR2. HIOECs were stimulated with *P. gingivalis* LPS at 1 µg/mL for 24 h. NSA at 2 or 4 µM was applied for 2 h before LPS stimulation. **a, b**. Western blots of TLR2 and necroptosis-associated proteins and bar graphs of relative fold changes of Western blots. **c, d**. NSA at 4 µM was applied for 2 h before LPS stimulation, then HIOECs were stimulated with *P. gingivalis* LPS at 1 µg/mL for 48 h. Expression of MLKL in red fluorescence and bar graphs of relative fold changes of MLKL. Scale bars, 50 µm. *, Significant difference compared to the control group. #, Significant difference compared to the LPS group. *, *P* < 0.05. #, *P* < 0.05. ##, *P* < 0.01.

**Figure 3.**
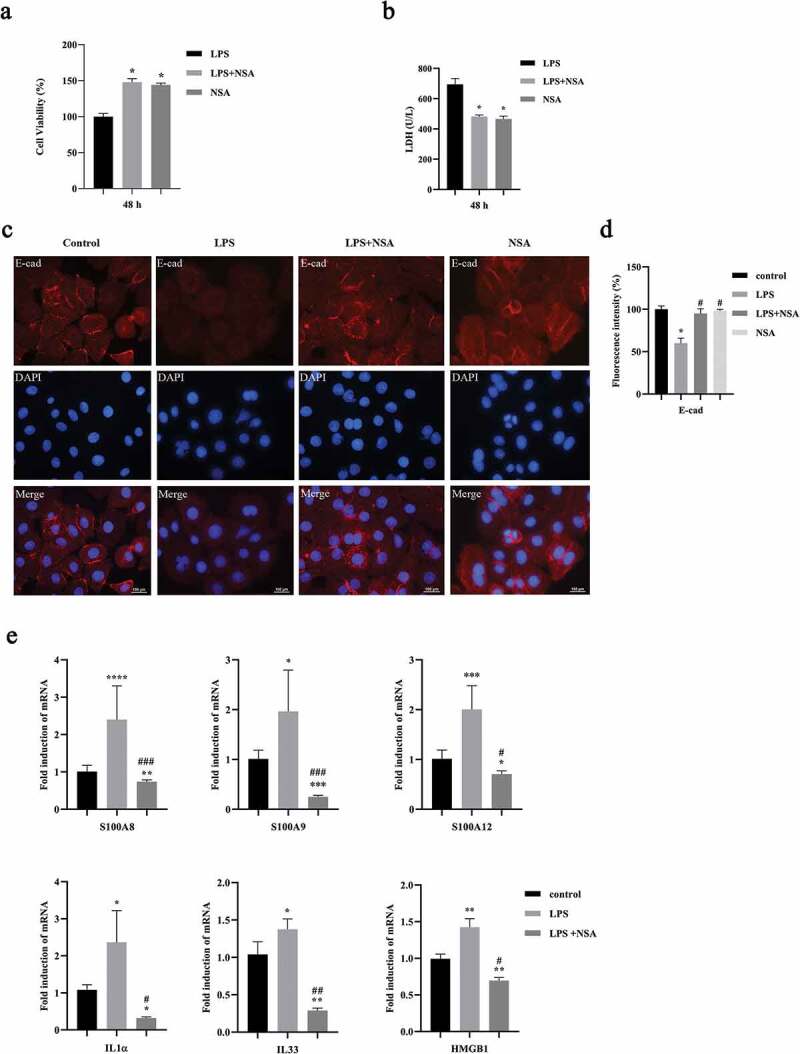
NSA blocked cell death, destruction of epithelial connection as well as DAMPs expression. HIOECs were stimulated with *P. gingivalis* LPS at 1 µg/mL for 24 h. NSA at 4 µM was applied for 2 h before LPS stimulation. **a, b**. Data of cell viability and LDH release with or without NSA. **c, d**. Effect of necroptosis on oral epithelial connection by E-cadherin immunofluorescence staining and bar graph. Red fluorescence indicates E-cadherin protein. Scale bars, 100 µm. **e**. Gene expression profiles of DAMPs by qPCR. *, Significant difference compared to the control group. #, Significant difference compared to the LPS group. *, *P* < 0.05. **, *P* < 0.01. ***, *P* < 0.001. ****, *P* < 0.0001.#, *P* < 0.05. ##, *P* < 0.01. ###, *P* < 0.001.

NSA is a specific inhibitor of MLKL, and was used to evaluate if NSA could inhibit necroptosis of HIOECs. The concentrations of NSA were determined by cell viability assay (Figure S1). It was demonstrated that NSA decreased the expressions of RIPK3, p-RIPK3, MLKL and p-MLKL compared to the *P. gingivalis* LPS group, while TLR2 retained the same as the control group (Figure 2a, b). Data from the immunofluorescence assay also showed that NSA significantly decreased the expression of MLKL (weaker red positive signals) compared to the *P. gingivalis* LPS group (Figure 2c, d). Meanwhile, LDH was also significantly decreased and the cell proliferation was increased in the *P. gingivalis* LPS plus NSA group while only the *P. gingivalis* LPS group resulted in decrease of the cell proliferation and increase of LDH (Figure 3a, b).

Loss of membrane integrity and release of intracellular DAMPs are regarded as typical properties of necroptosis [29]. We wanted to evaluate if the necroptosis induced by *P. gingivalis* LPS affects epithelial cell connections. *P. gingivalis* LPS decreased the expression of E-cad while the NSA could retain the expression of E-cad in the HIOECs compared to the control group (Figure 3c, d). Next, we found that *P. gingivalis* LPS could increase expressions of DAMPs, including S100A8, S100A9, S100A12, IL1α, IL33 and HMGB1 in HIOECs while NSA decreased these expressions (Figure 3e).

### Effects of oral epithelial cells-derived DAMPs on Mφ polarization

**Figure 4.**
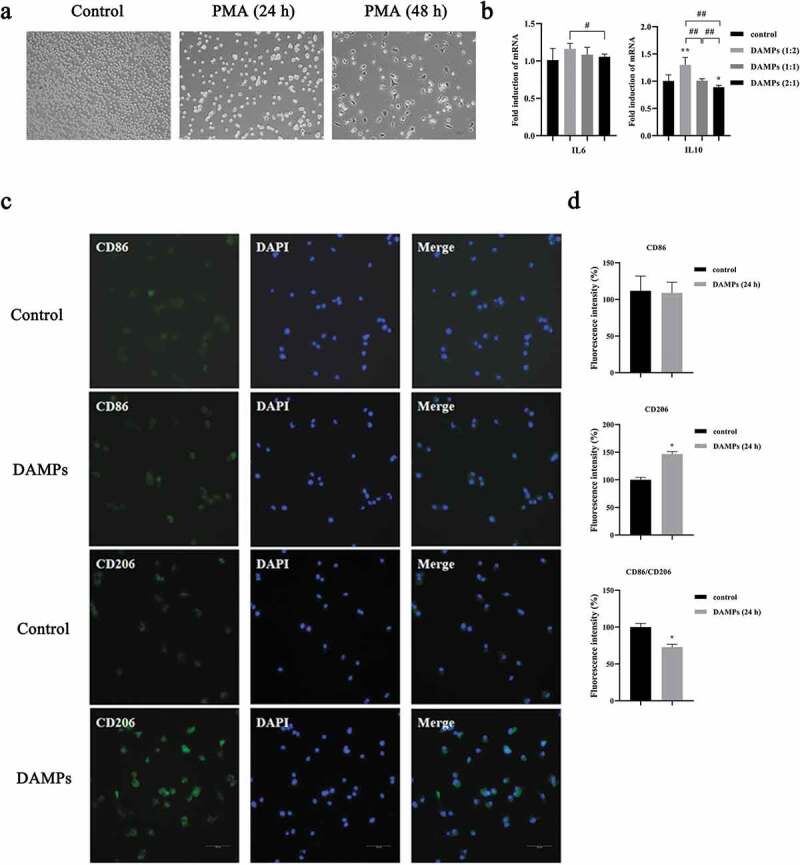
Low dose of oral epithelial cells-derived DAMPs promoted M2 polarization. **a**. Cell images of U937 cells cocultured with PMA at different time points. Scale bars, 200 µm. **b**. Gene expressions of IL6, and IL10 in M0 Mφs by qPCR. *, Significant difference compared with the control group. #, Significant difference compared with different groups. *, *P* < 0.05. **, *P* < 0.01. #, *P* < 0.05. ##, *P* < 0.01. **c**. M0 Mφs were stimulated with DAMPs at 1:2 dose, then CD86 and CD206 were immunofluorescence stained with and without DAPIs. **d**. Bar graphs of CD86 and CD206 immunofluorescence staining by fluorescence intensity with the Image J software (1.42q). *, *P* < 0.05.

During tissue damage, cellular stress, and necrotic cell death such as necroptosis, intracellular alarm signals are induced as DAMPs to eliminate pathogens and to activate repair mechanism [30]. As known, DAMPs released from necroptotic cells are immunomodulatory factors. To verify the successful preparation of DAMPs from HIOECs, we first tested cytokines expression stimulated by DAMPs in Mφs [14]. U937 cells cultured with PMA for 48 h was considered as the M0-like phenotype of Mφ (Figure 4a) [26]. Then, we evaluated the effect of HIOECs-released DAMPs with various doses on the gene expression of pro-inflammatory cytokine, IL6 and anti-inflammatory cytokine, IL10 (Figure 4b). Though data from qPCR assays showed that no significantly different expressions of IL6 can be seen when M0-like Mφs were cultured with DAMPs compared to the control group, DAMPs at the 1:2 dose significantly induced the expression of IL10, which clearly indicated the immunomodulatory effect of DAMPs prepared from HIOECs. The interesting results about the possible anti-inflammatory role of DAMPs at a low dose prompted us to further evaluated if DAMPs from HIOECs could modulate Mφ polarization. Data demonstrated that CD206 positive M2 Mφs with stimulation of DAMPs at 1:2 dose were significantly increased compared to control, and the ratio of M1/M2 was accordingly decreased (Figure 4c, d).

**Figure 5.**
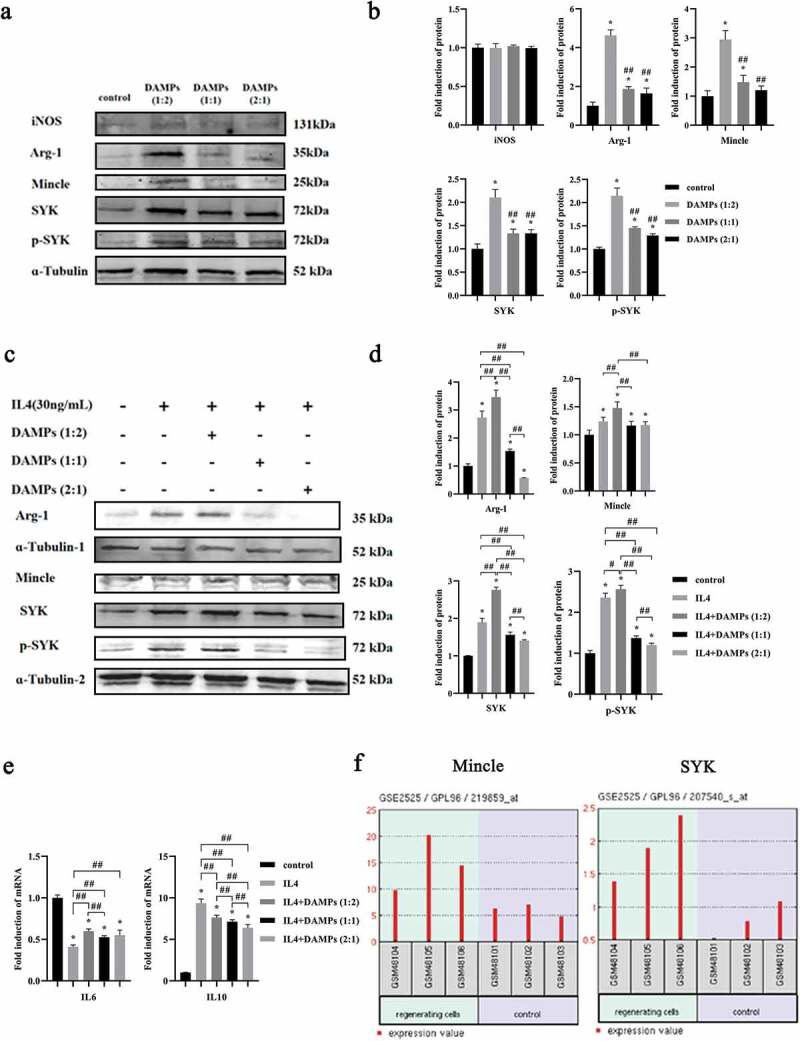
Effects of various doses of oral epithelial cells-derived DAMPs on Mφ polarization. M0 of M2 Mφs were stimulated with DAMPs at various doses for 24 h. **a, b**. Western blots of iNOS, Arg-1, Mincle, SYK, p-SYK and ɑ-Tubulin expressed by M0 Mφs and bar graphs of relative fold change compared by the normalization to ɑ-Tubulin. *, Significant difference compared with the control group. #, Significant difference compared to DAMPs (1:2) group. **c, d**. Western blots of Arg-1, Mincle, SYK, p-SYK and ɑ-Tubulin and bar graphs of relative fold change. **e**. Gene expressions of IL6, and IL10 in M2 Mφs by qPCR. *, Significant difference compared with the control group. #, Significant difference compared with different groups. *, *P* < 0.05. ##, *P* < 0.01. **f**. Relative expressions of Mincle/SYK in regenerated cells and normal cells in gingival tissues (GSE2525).

To further confirm the occurrence of Mφ polarization, the marker of the M1 phenotype, iNOS and the marker of the M2 phenotype, Arg-1 were evaluated by Western blot after M0-like Mφs were cocultured with DAMPs at various doses. Expression of the M2 marker, Arg-1 was dramatically increased when Mφs were cultured with DAMPs at 1:2 dose compared to the control group (Figure 5a, b) but significantly decreased in groups of DAMPs at 1:1 and 2:1 compared to the group of DAMPs at 1:2 dose (Figure 5a, b). Interestingly, expression of the M1 marker, iNOS did not show significantly difference at any dose of DAMPs compared to the control group (Figure 5a, b). Herein, we evaluated the possible effect of DAMPs on M2-like Mφs. IL4 is a well-known pleiotropic anti-inflammatory cytokine, which can induce M2-like Mφs [18]. Our data demonstrated that DAMPs at 1:2 dose significantly increased the expression of Arg-1 in M2-like Mφs. However, the expression of Arg-1 was dramatically decreased as the doses of DAMPs increased compared to the IL4 group (Figure 5c, d). We also tested the cytokines expression in M2-like Mφs stimulated by DAMPs (Figure 5e). Various doses of DAMPs promoted the expression of IL6 and inhibited the expression of IL10 compared to the IL-4 induced M2-like Mφs group (Figure 5e). Specifically, the expression of IL10 in the M2-like Mφs was continuously downregulated with increase of the DAMPs (Figure 5e).

In short, our results indicated that a relatively low dose of DAMPs (1:2) could induce the M2 phenotype polarization in M0-like Mφs as well as M2-like Mφs. However, on the contrary, such repair effect on the M2 polarization was inhibited especially in M2-like Mφs with increased release of DAMPs modulated by necroptosis.

### The Mincle/SYK axis was involved in Mφs polarization regulated by DAMPs

Mincle is one of the PRRs mainly expressed in inflammatory Mφs. To date, the role of Mincle/SYK in periodontitis is unclear. Our expression data from Western blots showed that DAMPs at 1:2 dose significantly induced expressions of Mincle, SYK and p-SYK in M0-like Mφs compared to the control group, which was similar to Arg-1 expression, while DAMPs at 1:1 and 2:1 dose had less effects (Figure 5a, b). Additional IL4 inducible assays demonstrated that M2-like Mφs (IL4) showed significant expressions of Mincle, SYK and p-SYK compared to the control. As expected, DAMPs at the 1:2 dose had similar promoting effects (Figure 5c, d). Meanwhile, M2-like Mφs cultured with higher doses of DAMPs (1:1 and 2:1) showed decreased expressions of Mincle, SYK and p-SYK compared to the lower dose of DAMPs (1:2) (Figure 5c, d).

### The expression of Mincle/SYK in regenerative cells of periodontal tissues

For the reason that Mφ polarization is a central event in both destructive and regenerative phases of periodontal disease, we tried to understand if Mincle/SYK was closely related to periodontal regeneration [19,31]. Fortunately, we have many publicly available databases which we can use to analyze our own interesting subject. Herein, we used the GEO database from NCBI to further analyze the relationship between Mincle/SYK and periodontal tissues. Analyzed data clearly demonstrated that expressions of Mincle and SYK were significantly higher in regenerative cells of periodontal tissues compared to normal cells (Figure 5f).

## Discussion

The most common oral diseases, periodontitis is a chronic inflammatory disease resulting in destruction of tooth supporting tissues. Oral epithelial tissue is the first physical and immunological barrier against periodontal infections. Our study demonstrated that RIPK3/MLKL-mediated necroptosis in oral epithelial cells was regulated by *P. gingivalis* LPS via TLR2. Necroptosis is a type of cellular death with massive release of DAMPs [32]. Our data further revealed that the anti-inflammatory effect of DAMPs sensed by Mincle/SYK was inhibited with its massive accumulation.

Recently, a new finding is that necroptosis is regulated by *P. gingivalis*, and involved in the destruction of periodontal supporting tissues, such as monocytes and PDLFs [10]. However, the role of *P. gingivalis* in oral epithelial cells, the first defensive cells against periodontal infection, is unclear. Noticeably, PDLFs have much lower tendency to necroptosis compared to monocytes during *P. gingivalis* challenge [10], which indicates that necroptosis regulated by *P. gingivalis* is different from various cellular origins. Our data clearly demonstrated that *P. gingivalis* LPS regulated necroptosis of oral epithelial cells leading to destruction of the oral epithelial barrier.

LPS, one of major virulence factors of *P. gingivalis*, is recognized by TLR2 or TLR4 [33]. Our data indicated that *P. gingivalis* LPS manipulated necroptosis in the oral epithelial cells by mainly activating TLR2, which is similar to a previous study [33,34]. We demonstrated that NSA, an inhibitor of MLKL, blocked not only expressions of RIPK3 and MLKL, which relate to necroptosis but also the expression of TLR2 as well as DAMPs that could be released from necroptotic cells. HMGB1 (high mobility group box 1), one of the DAMPs, can be recognized by TLR2, and is decreased with the inhibition of necroptosis [35]. We also found that *P. gingivalis* LPS-induced HMGB1 expression and was decreased when applied with NSA. Our data clearly suggest that *P. gingivalis* LPS activates TLR2 and binds to TLR2 resulting in necroptosis and release of DAMPs, and the released DAMPs may also bind to TLR2 and promote further destruction.

In this study, NSA also rescued the decreased expression of E-cad induced by *P. gingivalis* LPS. E-cad is one of the major transmembrane proteins of the adherence junction between neighboring epithelial cells. To date, a number of studies focused on the role of *P. gingivalis* in adherence junction of the oral epithelial barrier [36–38]. However, the underlying mechanisms still remain unclear. It was reported that the cleavage of E-cad from the epithelial cell surface was regulated by MLKL-dependent necroptosis [39]. Our results indicated necroptosis as a novel mechanism of destroyed epithelial connection induced by *P. gingivalis* LPS but further studies are still needed.

To date, NSA has been investigated as a powerful inhibitor of necroptosis in various disease models [9,11,40]. Specific to periodontitis, it was reported that intraperitoneally treatment of NSA in a *P. gingivalis*-soaked ligatures induced animal model attenuated alveolar bone loss induced by *P. gingivalis* [9]. However, the exact therapeutic target for the application of NSA in periodontal therapy is still unclear. In this study, NSA was currently used to block necroptosis of oral epithelial cells. Thus, further studies of animal models with periodontitis should be made to evaluate the effect of NSA by the epithelial route of administration within the oral cavity, which may be more instructive to clinical application.

Periodontitis is a type of infectious disease initiated by dental plaque biofilm. Therefore, periodontal pathogens are commonly regarded as the major reason. However, recent studies reveal that endogenous DAMPs play a critical role in further damage of periodontal tissues [41,42]. For instance, anti-HMGB1 neutralizing antibody inhibited periodontal inflammation and bone resorption in an animal periodontitis model [41]. DAMPs, S10012 from the S100 subfamily is increased in inflamed gingival tissues [42]. This indicates the important or modulative roles of DAMPs in pathogenesis and treatment of periodontitis. From a different perspective, we focused on a possible role of DAMPs released by oral epithelial cells in the regulation of Mφ polarization. Our data indicated that a low dose of DAMPs (1:2) induced the polarization from M0 towards the M2-like phenotype. This phenomenon, however, was obviously weakened with increase of DAMPs. Further, M2 polarization was inhibited by increasing doses of DAMPs (1:1 and 2:1) in the IL-4 induced M2-like Mφs. DAMPs are the alarmins signals which are considered as dangerous signals released by dead cells [43]. In the present study, we confirmed that the limited dose of DAMPs alarmins sensed by Mincle/SYK induced self-repair mechanism by promoting the polarization towards the M2-like phenotype in order to produce cytokines, such as IL-10. However, as the dose of ‘alarmins’ increased, the M2 polarization was inhibited especially in M2-like Mφs. Hence, though no significant M1 phenotype maker can be seen under DAMPs stimulation, the ratio of the M1/M2 phenotype Mφs would be comparatively increased as a consequence, which suggested that massive-accumulation of DAMPs resulted in the exacerbation of inflammation and the eventual damage of periodontal tissues.

Mincle, which is one of the C-type lectin-like receptors (CLRs), and mainly expressed by Mφs, is reported to sense DAMPs, such as nuclear SAP130 and promote inflammatory diseases [44]. It has been shown that Mincle plays an essential role in maintaining the M1 Mφs phenotype [45]. However, it was reported that the activation of Mincle resulted in the production of IL10 [46]. The anti-inflammatory role of Mincle/SYK was also revealed under infection with *Helicobacter pylori* [47]. In this study, we tried to explore the possible effect of Mincle/SYK on Mφs polarization. Our data showed that Mincle/SYK were expressed by M0-like and M2-like Mφs leading to simultaneously up-regulation of Arg-1 and IL10 (M2 marker) in response to DAMPs (at a dose of 1:2). We supposed that the composition of DAMPs might vary among different doses. With low doses of DAMPs, certain DAMPs might present effective concentrations to promote M2 polarization. Thus, future studies to reveal specific DAMPs from oral epithelial cells in the activation of Mincle will help understand the exact role of Mincle/SYK in Mφs polarization.

Due to the role of the M2 phenotype Mφs in anti-inflammation, it was known that M2-like Mφs polarization in the early stage of tissue repair promoted periodontal regeneration after transplantation with stem cells [31]. Interestingly, we also found overexpression of Mincle/SYK in the regenerative cells compared to control cells. These results indicate that the anti-inflammatory role of Mincle is consistent with previous publications where Mincle participates in the production of anti-inflammatory cytokines and in the inhibition of pro-inflammatory signals [22,24,25,48]. To our knowledge, this is the first study to reveal the anti-inflammatory role of Mincle/SYK in the regulation of Mφ polarization in periodontitis although more studies are still needed to investigate the underlying mechanisms.

**Figure 6.**
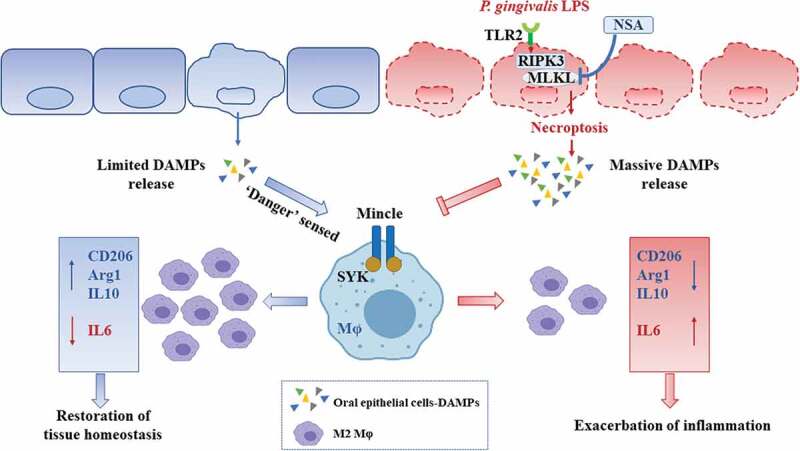
Diagram of proposed schematic. Within a periodontal infection microenvironment, the limited accumulation of DAMPs which might be induced by cell death such as apoptosis are sensed as ‘alarming’ by PRRs including Mincle and therefore facilitates M2 polarization for the restoration of tissue homeostasis. However, *P. gingivalis* LPS manipulates RIPK3/MLKL-mediated necroptosis via TLR2 in oral epithelial cells. The massive DAMPs released from oral epithelial cells induced by necroptosis inhibit M2 polarization by inactivating the Mincle/SYK signal. NSA, the inhibitor of MLKL might be applied to alleviate the effect of necroptosis on oral epithelial destruction as well as to block the inhibition of M2 polarization induced by massive DAMPs.

In conclusion, our data reveal that necroptosis induced by *P. gingivalis* LPS within the periodontal microenvironment results in the impaired oral epithelial integrity by continuous activation of TLR2. Massive DAMPs released by oral epithelial cells inhibit M2 polarization via Mincle/SYK signals and Mincle can be a promising target for the prevention and treatment of periodontitis (Figure 6). To inhibit necroptosis of oral epithelial cells, NSA will block oral epithelial destruction as well as massive accumulation of DAMPs at the source. In the future, we will try to further understand specific DAMPs in the regulation of oral epithelial barriers as well as in Mφs activation.

## Supplementary Material

Supplemental Material
