# Supplementary material for: Porphyromonas gingivalis lipopolysaccharide induced RIPK3/MLKL-mediated necroptosis of oral epithelial cells and the further regulation in macrophage activation
Source: J Oral Microbiol. 2022 Feb 27;14(1):2041790. doi: 10.1080/20002297.2022.2041790 (PMC8890547; doi:10.1080/20002297.2022.2041790)
Supplement: Supplemental Material [file ZJOM_A_2041790_SM2027.docx]

Supplementary materials

**Table S1.** Primer sequences used in this study~~.~~

| S100A8 | Forward | ATGCCGTCTACAGGGATGAC |
| --- | --- | --- |
|  | Reverse | ACGCCCATCTTTATCACCAG |
| S100A9 | Forward | CAGCTGGAACGCAACATAGA |
|  | Reverse | TCAGCTGCTTGTCTGCATTT |
| S100A12 | Forward | CGGAAGGGGCATTTTGACACC |
|  | Reverse | CCTTCAGCGCAATGGCTACC |
| IL1α | Forward | CCGTGAGTTTCCCAGAAGAA |
|  | Reverse | ACTGCCCAAGATGAAGACCA |
| IL33 | Forward | AGCAAAGTGGAAGAACACAGC |
|  | Reverse | CTTCTTTGGCCTTCTGTTGG |
| HMGB1 | Forward | TGTAAGGCTGTGTAAGATT |
|  | Reverse | AAGGTTAGTGGCTATTGAA |
| IL-6 | Forward | AAGCCAGAGCTGTGCAGATGAGTA |
|  | Reverse | TGTCCTGCAGCCACTGGTTC |
| IL-10 | Forward | GAGATGCCTTCAGCAGAGTGAAGA |
|  | Reverse | AGTTCACATGCGCCTTGATGTC |

**Figure S1**


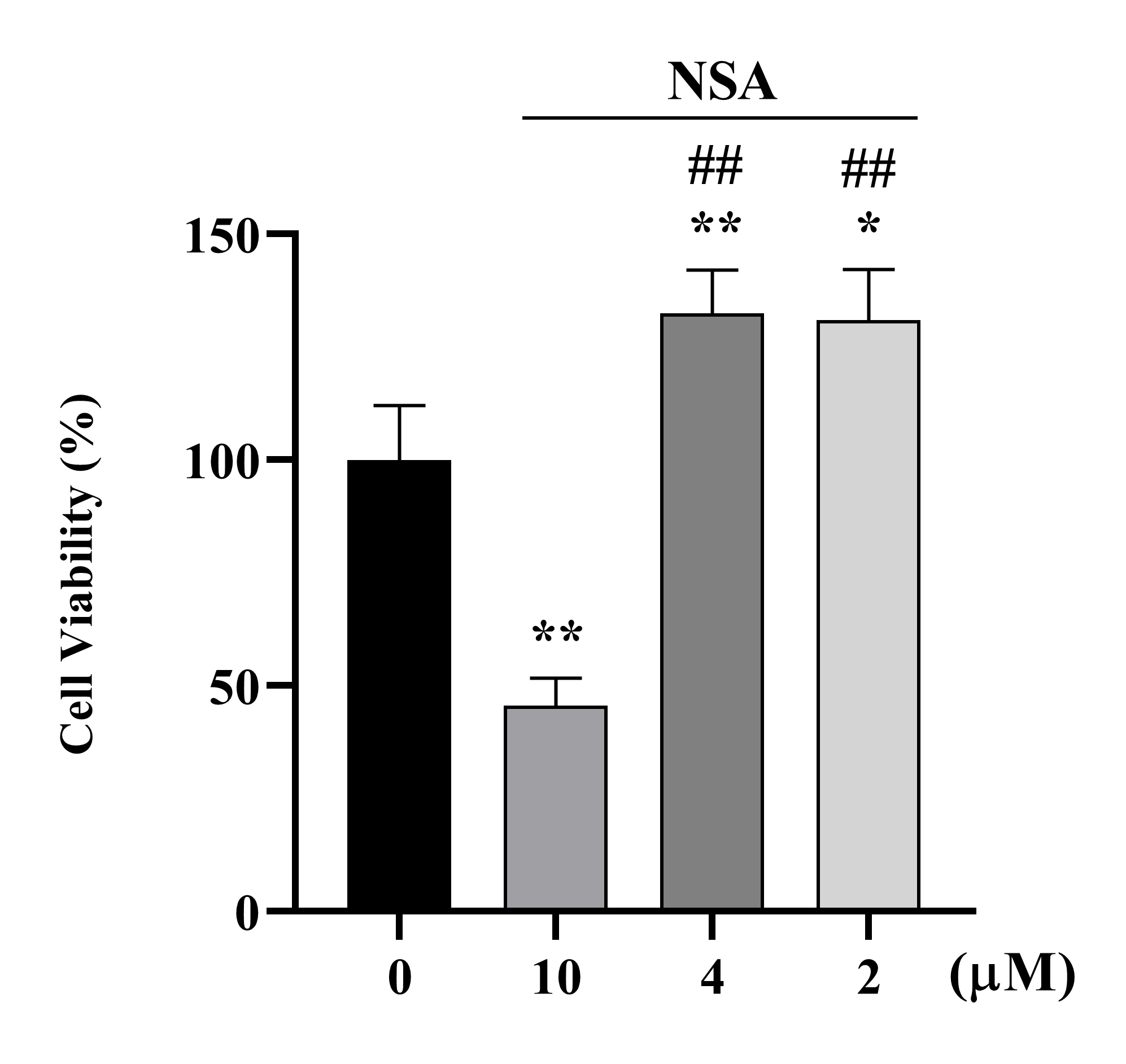


**Figure S1.** To confirm the concentration of NSA, the CCK8 assay was applied to test the effect of NSA on cytotoxicity. As shown, NSA at 10 μM significantly inhibited cell viability of HIOECs, while NSA at 4 and 2 μM had no cytotoxic effect on HIOECs. The cell viability of HIOECs were significantly increased at 4 and 2 μM. *, Significant difference compared to the 0 μM group. #, Significant difference compared to the 10 μM group. *, *P* < 0.05. **, *P* < 0.01. ##, *P* < 0.01.
